# Disruption of the GABAergic system contributes to the development of perioperative neurocognitive disorders after anesthesia and surgery in aged mice

**DOI:** 10.1111/cns.13388

**Published:** 2020-06-02

**Authors:** Wen Zhang, Bing‐Rui Xiong, Long‐Qing Zhang, Xian Huang, Wen‐Chang Zhou, Qian Zou, Anne Manyande, Jie Wang, Xue‐Bi Tian, Yu‐Ke Tian

**Affiliations:** ^1^ Department of Anesthesiology Tongji Hospital Tongji Medical College Huazhong University of Science and Technology Wuhan China; ^2^ Department of Anesthesiology Zhongnan Hospital Wuhan University Wuhan China; ^3^ Department of Physiology School of Basic Medicine and Tongji Medical College Huazhong University of Science and Technology Wuhan China; ^4^ Department of Radiology Tongji Hospital Tongji Medical College Huazhong University of Science and Technology Wuhan China; ^5^ School of Human and Social Sciences University of West London London United Kingdom; ^6^ State Key Laboratory of Magnetic Resonance and Atomic and Molecular Physics Key Laboratory of Magnetic Resonance in Biological Systems Wuhan Center for Magnetic Resonance Wuhan Institute of Physics and Mathematics Chinese Academy of Sciences Wuhan China

**Keywords:** GABAergic system, mitogen‐activated protein kinase, neuroinflammation, perioperative neurocognitive disorders, α5GABA_A_ receptors

## Abstract

**Aims:**

Perioperative neurocognitive disorders (PND) are associated with cognitive impairment in the preoperative or postoperative period, and neuroinflammation is thought to be the most important mechanisms especially during the postoperative period. The GABAergic system is easily disrupted by neuroinflammation. This study investigated the impact of the GABAergic system on PND after anesthesia and surgery.

**Methods:**

An animal model of laparotomy with inhalation anesthesia in 16‐month‐old mice was addressed. Effects of the GABAergic system were assessed using biochemical analysis. Pharmacological blocking of α5GABA_A_Rs or P38 mitogen‐activated protein kinase (MAPK) were applied to investigate the effects of the GABAergic system.

**Results:**

After laparotomy, the hippocampus‐dependent memory and long‐term potentiation were impaired, the levels of IL‐6, IL‐1β and TNF‐α up‐regulated in the hippocampus, the concentration of GABA decreased, and the protein levels of the surface α5GABA_A_Rs up‐regulated. Pharmacological blocking of α5GABA_A_Rs with L655,708 alleviated laparotomy induced cognitive deficits. Further studies found that the P38 MAPK signaling pathway was involved and pharmacological blocking with SB203,580 alleviated memory dysfunctions.

**Conclusions:**

Anesthesia and surgery caused neuroinflammation in the hippocampus, which consequently disrupted the GABAergic system, increased the expressions of surface α5GABA_A_Rs especially through the P38 MAPK signaling pathway, and eventually led to hippocampus‐dependent memory dysfunctions.

## 1 INTRODUCTION

Perioperative neurocognitive disorders (PND), a general term for cognitive impairment identified during the preoperative or postoperative period, are known to negatively affect multiple cognitive domains such as memory, attention, and concentration.^1^, ^2^, ^3^ At the point of discharge, the incidence of PND is 25% to 40% among the elderly^4^ and significantly affects patients’ outcomes and increases mortality, especially in aging patients.^5^

Neuroinflammation is a common factor contributing to cognitive deficits especially the hippocampus‐dependent memory impairment.^5^, ^6^, ^7^, ^8^, ^9^ Neuroinflammation is also a dynamic, multi‐stage physiological response, mainly manifesting as the activation of natural immune cells in the central nervous system, accompanied by the release of a variety of pro‐inflammatory factors that ultimately lead to changes in homeostasis in the central microenvironment.^10^ However, the exact mechanism underlying how neuroinflammation causes memory deficits is not well understood and there are no treatments that are available to effectively reverse or prevent memory deficits after anesthesia and surgery.^11^ Therefore, it is necessary to explore the downstream mediators of neuroinflammation that induce memory deficits.

Changes in multiple neurotransmitter receptors have been demonstrated to be associated with memory deficits.^12^, ^13^ The GABAergic system also participates in the processes of learning, memory, and synaptic plasticity.^14^ GABA type A receptors (GABA_A_Rs) comprise different subunits, and different combinations of GABA_A_Rs have shown different localization and distinct physiological and pharmacological characteristics.^15^ In particular, the α5‐subunit‐containing subtype of GABA_A_Rs (α5GABA_A_Rs), which makes up 20%‐25% of the hippocampal GABA_A_Rs,^15^ are specifically localized to extrasynaptic regions of hippocampal pyramidal neurons and are mainly involved in mediating tonic inhibition, as well as being implicated in processing memory.^16^, ^17^ Furthermore, the increase in α5GABA_A_Rs activity causes profound memory blockade. Parallelly, a reduction in the expression or functions of the α5GABA_A_Rs improves certain memory performance.^14^, ^18^ Here, we hypothesized that anesthesia and surgery will cause neuroinflammation in the hippocampus, targeting the GABAergic system, especially the α5GABA_A_Rs pathway, affecting LTP and resulting in hippocampus‐dependent memory deficits.

## 2 MATERIALS AND METHODS

### 2.1 Animals

A total of 183 female c57BL/6J mice (16‐month‐old) were purchased from the Experimental Animal Center of Tongji Medical College, Huazhong University of Science and Technology. All animals were housed five per cage in maintained temperature of 22 ± 1°C with a 12‐hour light/dark cycle with free access to food and water. All procedures were in accordance with the Guidelines of the National Institutes of Health Guide for the Care and Use of Laboratory Animals.

### 2.2 Groups and Laparotomy surgery

The laparotomy model was established as previously described with minor improvements.^3^ Mice were inducted with 3% isoflurane and maintained with 1.3% isoflurane. Then, an incision about 1.0 cm was made at the site 0.5 cm below the right rib. The small intestine of about 10 cm was exposed onto a sterile gauze for 15 minutes and then returned back into the abdominal cavity. The muscle and skin were closed with 4‐0 sutures, respectively. Lidocaine cream was applied at the incision site to reduce postoperative pain. For the anesthesia group, mice only received anesthesia as described above, while for the control group, mice were given oxygen in the induction box with free movement.

### 2.3 Novel object recognition test

The operator was blinded to the experiment and handled the mice for 1 minute a day, for a total of 6 days before the test. Then, mice were put into the box to accommodate to the condition for 5 minutes. In the training stage, two identical rectangular blocks were placed on the same side of the box, and the mice were allowed to explore for 5 minutes. Exploratory behaviors included sniffing, licking, and climbing on pieces of wood. In the testing stage, a rectangular block was replaced by a cylinder, and mice were placed into the box to explore for another 5 minutes. The learning and memory ability were evaluated by the discrimination ratio which is represented by C/(A + C), where C is the time spent exploring the novel object, A is the time spent exploring the familiar object, and A + C is the total time spent exploring the two objects. In addition, the mice were screened when the total exploring time was less than 5 seconds or they explored only one of the objects during the training phase.

### 2.4 Fear condition test

Fear condition test (FCT) was performed as previously reported.^3^ Briefly, after mice accommodated to the condition, one tone‐foot‐shock pairing was given (tone, 30 seconds, 70 dB, 1 kHz; foot‐shock, 2 seconds, 0.5 mA, a 30‐second interval after the shock). Then, they were given another shock pairing (three pairings in total). 24 hours after the training session, the mice were put back into the same test chamber to assess the contextual fear conditioning. Two hours later, the tone fear conditioning was assessed. Mice were placed into a novel chamber that changed the environment, and the same tone was delivered for 3 minutes. Freezing behavior was defined as the absence of all visible movement except for respiration.

### 2.5 Nuclear magnetic resonance

Brain tissues for nuclear magnetic resonance (NMR) analysis were performed as previously conducted^19^ and briefly described as following. In order to avoid the impact of postmortem changes, mice were deeply anesthetized with 4% isoflurane and then microwaved using a domestic microwave oven (0.75 kw, 15 seconds). After that, brain tissue was taken, weighed, and quickly frozen to −80°C.

HCl/methanol (200 μL, 0.1 mol/L) and 60% ethanol (vol/vol, 400 μL) were added into the EP tubes and homogenized with Tissuelyser for 90 seconds at a frequency of 20 Hz (TissuelyserⅡ, QIAGEN, Germany). The mixture was centrifuged for 15 minutes at 14000 g, and the supernatant was collected into a 5‐mL EP tube. The substance was extracted twice with 800 μL 60% ethanol. All the supernatants were collected and desiccated with the centrifugal drying apparatus (Thermo Scientific 2010), and the dried product was collected for further NMR studies.

The phosphate buffer solution (PBS, pH = 7.2, 60 μL, 120 mg/L 3‐[trimethylsilyl] propionic‐2, 2, 3, 3, d4 acid sodium salt [TSP, 269913‐1G; Sigma‐Aldrich] in D2O) and the double‐distilled water (540 μL) were added into the 5‐mL EP tubes to dissolve the dried product, and TSP was set as the internal standard. The solution was shaken evenly with a high‐speed vortex until the precipitates were dissolved, and the mixture centrifuged at 14000 g for 10 minutes. The supernatant (530 μL) was then collected and transferred to a 5‐mm NMR tube for 1H NMR analysis.

NMR spectra testing were performed at 298 K on a Bruker Avance III 600 MHz NMR spectrometer equipped with an inverse cryogenic probe (Bruker Biospin, Germany). The 1H NMR spectra were acquired with a standard WATERGATE pulse sequence and processed in the commercial software TOPSPIN and NMRSpec, as well as a home‐made tool based on a MATLAB code.

### 2.6 MSD multi‐spot assay

The hippocampus was homogenized and centrifuged at 14000 g  for 15 minutes at 4°C. The supernatants were collected, and the levels of IL‐6, IL‐1β, and TNF‐α were detected using commercially available proinflammatory panel 1 (mouse) kits (Meso Scale Discovery [MSD®]).^20^ The procedures were performed according to the manufacturer's instructions, and the concentrations of IL‐6, IL‐1β, and TNF‐α are presented as pg/mL.^8^

### 2.7 Electrophysiology in vitro

Mice were deeply anesthetized with pentobarbital sodium (50 mg/kg, *i.p*.) and then decapitated. The brain was quickly removed and placed into an ice‐cold oxygenated (95% O_2_ and 5% CO_2_) high‐sucrose solution that contained (in mmol/L): 213 sucrose, 3 KCl, 1 NaH_2_PO_4_, 0.5 CaCl_2_, 5 MgCl_2_, 26 NaHCO_3_, and 10 glucose. Hippocampal slices (300‐320 μm) were prepared as described previously.^21^, ^22^, ^23^ The slices were transferred to a holding chamber containing ACSF consisting of (in mmol/L): 124 NaCl, 26 NaHCO_3_, 3 KCl, 1.2 MgCl_2_·6 H_2_O, 1.25 NaH_2_PO_4_·2 H_2_O, 10 C_6_H_12_O_6_, and 2 CaCl_2_ at PH 7.4, 305 mOsm. The slices were allowed to recover at 31.5°C for 30 minutes and then at room temperature (RT) for at least 1 hour.

Acute slices were transferred to the recording chamber, and the long‐term potentiation (LTP) of evoked field postsynaptic potentials (fPSPs) was recorded from the stratum radiatum in CA1 following electrical stimulation of the Schaffer collateral pathway. After the stable baseline of at least 30 minutes, high‐frequency stimulation (HFS, 100 Hz, 50 pulse, four trains at 20‐second interval) was used to induce LTP and then recorded for another 60 minutes.

### 2.8 Western blot

Hippocampal protein samples were prepared as previously described^24^ and were separated using 10% SDS‐PAGE and subsequently transferred to polyvinylidene fluoride membranes (Millipore) for electroblotting. The membranes were blocked with 5% BSA in TBST (0.1%) for 2 hours at RT, incubated with primary antibody overnight at 4°C, and then incubated with horseradish peroxidase (HRP)‐conjugated secondary antibodies for 2 hours at RT. The antibodies used in this study include rabbit anti‐α5GABA_A_ receptors, anti‐GAT‐3 (1:500‐1000; Alomone Labs), rabbit anti‐GAD65 (1:1000; Abcam), rabbit anti‐P38, p‐P38, ERK1/2, p‐ERK1/2, JNK1/2, p‐JNK1/2 (1:1000‐2000; Cell Signaling Technology), mouse anti‐GAPDH HRP‐conjugated goat‐anti‐mouse IgG, or anti‐rabbit IgG (1:1000‐5000; Promoter). The protein bands were visualized using chemiluminescence (Pierce ECL Western Blotting Substrate; Thermo Scientific) and measured using a computerized image analysis system (ChemiDoc XRS+; BIO‐RAD).

### 2.9 Immunofluorescence

Brain slices for immunofluorescence were prepared as previously reported.^24^ The sections were blocked with 10% donkey serum and 0.3% Triton 1 hour at RT. Then, the sections were incubated overnight at 4°C with mouse anti‐Iba1 antibody (1:300; Wako). After washing with PBS, the sections were incubated with Alexa Fluor 488‐labeled donkey anti‐rabbit secondary antibody (1:200; Invitrogen) at 37°C for 2 hours. Images were captured using a laser scanning confocal microscope (FV1000; Olympus).

### 2.10 Quantitative real‐time PCR

Total RNA and cDNA from the hippocampus were prepared as outlined before.^3^ Quantitative real‐time PCR (RT‐PCR) was performed on the ABI7900 (Illumina) with SYBR Green Master Mix kiTAKARA). The conditions for the PCR were as following: incubated at 50°C for 2 minutes and then at 95°C for 10 minutes and then followed by 40 cycles at 95°C for 30 seconds and 60°C for 30 seconds. The sequences of specific primers are summarized in Table 1.

**Table 1:** The sequence of primers for RT‐PCR analysis

Name	Primer	Sequence(5ʹ‐3ʹ)	Size
Mus α5 subunit	Forward	ATGACCCAAACCCTCCTTGT	164 bp
	Reverse	CGCAGTCTGTTGTCATAGCC	
Mus α1 subunit	Forward	AGAGAGGGTATGCGTGGGA	219 bp
	Reverse	TTTTCTTGGGTTCTGGTGG	
Mus β3 subunit	Forward	ACCATGACAACCATCAACAC	100 bp
	Reverse	ATACAAAGACAAAGCAGCCC	
Mus GAPDH	Forward	ATGGGTGTGAACCACGAGA	229 bp

### 2.11 Statistical analysis

All results are presented as mean ± SEM. An unpaired Student's t‐test was used to compare two groups. For three groups, one‐way ANOVA followed by Bonferroni post hoc test was applied. Two‐way ANOVA was used to analyze Novel object recognition test (NORT) and FCT after using L655,708 or SB203,580. The experimental data all conform to the normal distribution. GraphPad Prism 7.0 was used for all analyses, and *P* < .05 was considered statistically significant in this study.

## 3 RESULTS

### 3.1 Hippocampus‐dependent memory and LTP were impaired after anesthesia and surgery in aged mice

**Figure 1:**
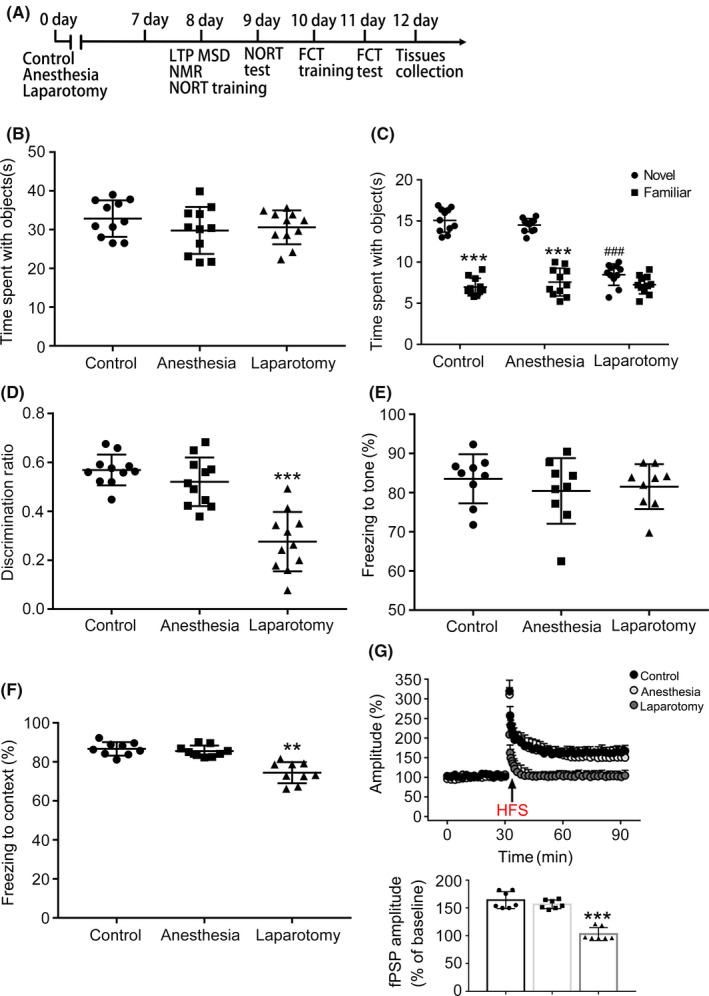
Behavioral tests and hippocampal LTP in aged mice. A, Illustration of the experimental processes. 16‐month‐old female mice were randomly divided into 3 groups (control, anesthesia and laparotomy). Behavioral tests were conducted from 8 days to 11 days after anesthesia or laparotomy. Samples were taken for LTP, MSD, and NMR 7 days after anesthesia or laparotomy. B‐D, In the NORT, the total time spent with two same objects was similar among the three groups. In the laparotomy group, the mice spent less time on the novel object and presented lower discrimination ratio compared with the other two groups (n = 11). E‐F, In the FCT, the mice in the laparotomy group showed lower freezing time to the context, and there was no difference in the tone freezing time (n = 11). G, Hippocampal LTP was impaired in the laparotomy mice (n = 7). Data are presented as mean ± SEM. ^**^ *P* < .01, ^***^ *P* < .001, ^###^ *P* < .001

In the NORT, no difference was found in the total time spent on identical objects among the three groups during the training stage (F_(2,30)_ = 1.07, *P* = .35; Figure 1B). In the testing phase, mice spent more time on the novel object than on the familiar object in the control and anesthesia‐treated groups (F_(2,40)_ = 147.7, *P* < .001; Figure 1C). However, the time spent on the novel and familiar objects did not differ in the laparotomy mice. Further analysis of the discrimination ratio revealed that there was a distinct difference among the three groups. And the discrimination ratio in the control and anesthesia groups was greater than that in the laparotomy group (F_(2,30)_ = 32.21, *P* < .001; Figure 1D). In the FCT, no statistical difference was found in tone freezing time which was the hippocampus‐independent memory (F_(2,30)_ = 1.29, *P* = .29; Figure 1E). However, there was a significant difference in the context freezing time among the three groups (F_(2,30)_ = 15.97, *P* < .01; Figure 1F). In this study, mice in the laparotomy group spent less freezing time than those in the control group, and there was no difference between the control and anesthesia groups (Figure 1F). Next, we assessed whether the hippocampal LTP was impaired after laparotomy. There was a remarkable increase in the amplitude of fPSP (% of baseline) in the control and anesthesia slices after HFS (F_(2,18)_ = 54.46, *P* < .001; Figure 1G). The amplitude was increased from 103.8% ± 2.6% to 164.1% ± 15.2% in slices from the control mice and 100% ± 0.7% to 156.5% ± 7.8% in the anesthesia slices. In contrast, LTP was impaired and increased slightly from 103% ± 2.4% to 103.3% ± 11.7% in the laparotomy slices (Figure 1G). These results demonstrate that deficits of hippocampus‐dependent memory and impairment of LTP were caused by anesthesia and surgery rather than by anesthesia alone.

### 3.2 Hippocampal neuroinflammation was observed after anesthesia and surgery in aged mice

**Figure 2:**
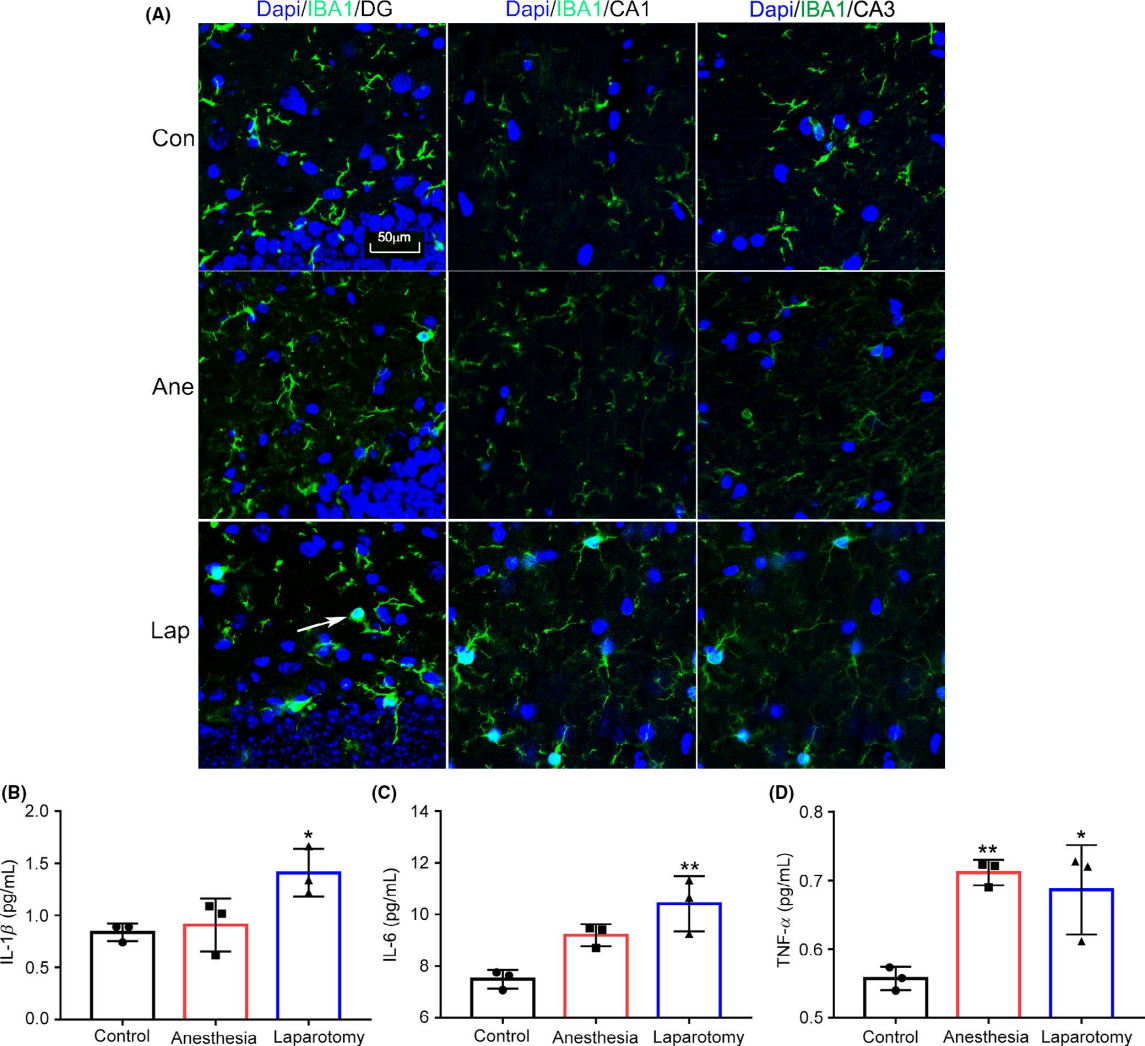
The morphology of microglia and the levels of inflammatory cytokines in the hippocampus. A, Microglia was activated in the CA1, CA3, and DG regions in the laparotomy mice. The white arrow points to the activated microglia. B‐D, The levels of IL‐1β, IL‐6, and TNF‐α in the laparotomy mice were up‐regulated, and TNF‐α was also increased in the anesthesia mice (n = 3). Data are presented as mean ± SEM. *P < .05, **P < .01

Compared with the control and anesthesia mice, the morphology of microglia in the laparotomy mice was clearly changed, and manifested mainly as hypertrophy in the cell body in the CA1, CA3 and DG regions of the hippocampus (Figure 2A). Next, we examined cytokine expressions of IL‐1β, IL‐6 and TNF‐α in the hippocampus. The MSD results showed that IL‐1β and IL‐6 were obviously up‐regulated (F_(2,6)_ = 7.05, *P* = .03; Figure 2B; F_(2,6)_ = 13.42, *P* = .006; Figure 2C) in the laparotomy group, but the expression of TNF‐α was increased both in the anesthesia and laparotomy groups (F_(2,6)_ = 12.7, *P* = .007; Figure 2D). These results demonstrate that anesthesia and surgery could cause severe inflammatory response in the hippocampus.

### 3.3 Hippocampal GABAergic system was disrupted, and surface α5GABA_A_Rs were selectively involved after anesthesia and surgery in aged mice

**Figure 3:**
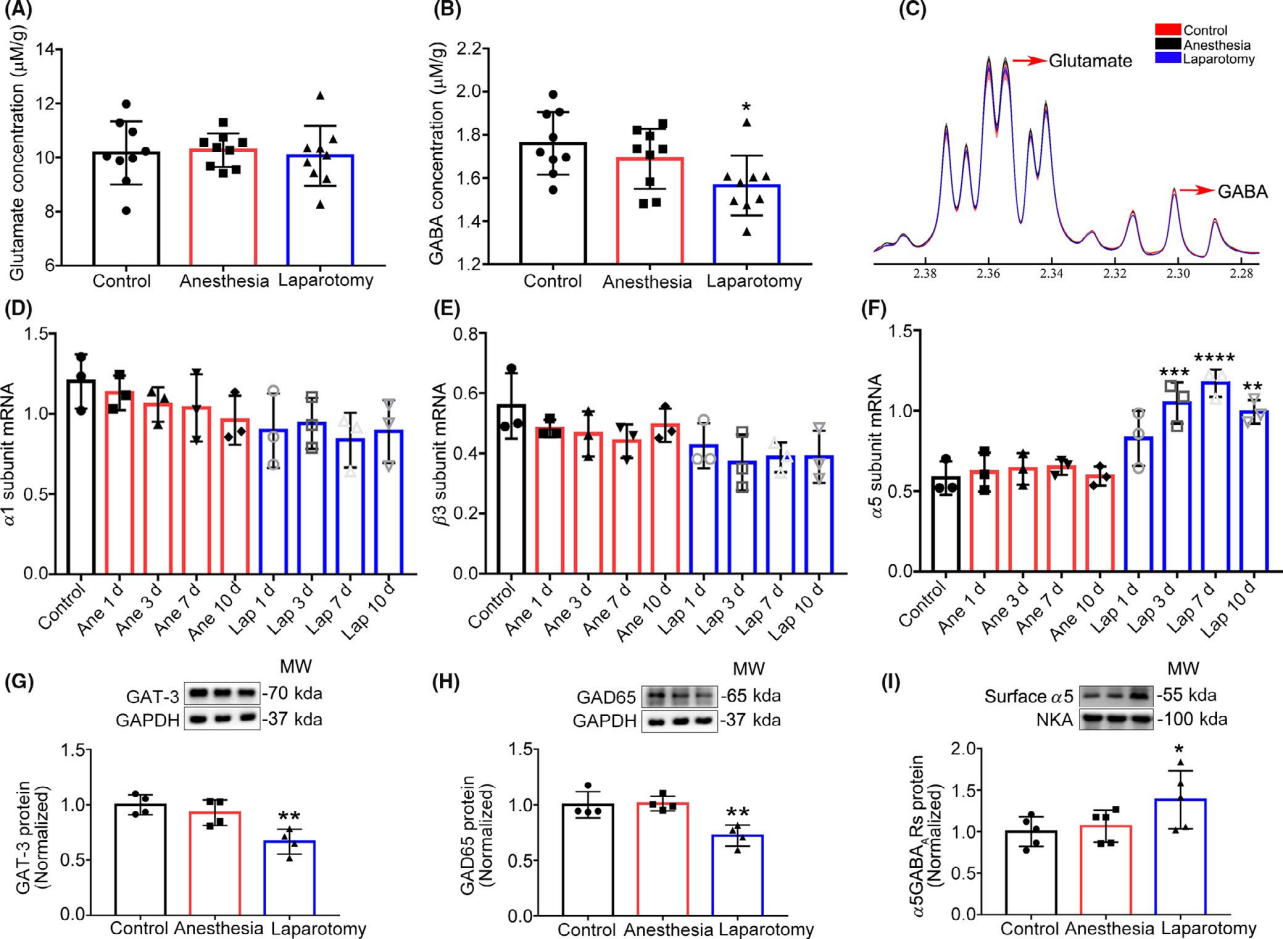
The expressions of neurotransmitters and different subunits of GABA_A_Rs. A‐B, The expression of GABA was decreased in the laparotomy mice, and no difference was found about glutamate (n = 9). C, The different average spectra of selected metabolites (GABA and glutamate). D‐F, The mRNA level of α5 subunit was up‐regulated at 1 day and continued to 10 days after laparotomy. No difference was found about the α1 and β3 subunits (n = 3). G‐I, The expressions of GAT‐3 and GAD65 were decreased, and the levels of surface α5GABA_A_Rs were increased in the laparotomy mice (n = 4). Data are presented as mean ± SEM. *P < .05, **P < .01, ***P<0.001, ****P<0.0001

Next, we examined the changes in levels of neurotransmitters after anesthesia and surgery in the hippocampus and used absolute concentrations to compare the differences among the three groups. The NMR results showed no difference in the levels of glutamate among the three groups (F_(2,24)_ = 0.11, *P* = .90; Figure 3A), while the levels of GABA were clearly decreased in the laparotomy group (F_(2,24)_ = 4.43, *P* = .02; Figure 3B). The raw data of the average and deviation of these two transmitters are presented (Figure 3C). Next, we examined the transcription levels of α5, α1, and β3 subunits, at 1, 3, 7, and 10 days after laparotomy using quantitative RT‐PCR. There was no significant difference at any time point of α1 (F_(8,18)_ = 1.49, *P* = .23; Figure 3D) and β3 (F_(8,18)_ = 2.05, *P* = .09; Figure 3E) subunit levels, while the α5 subunit level was increased at 1 day and continued to increase at 3, 7, and 10 days after laparotomy (F_(8,18)_ = 13.85, *P* < .0001; Figure 3F). Then, we detected the protein levels of GAT‐3, GAD65, and surface α5GABA_A_Rs using Western blot. The results showed that the expressions of GAT‐3 and GAD65 were evidently decreased after laparotomy (F_(2,9)_ = 10.82, *P* = .004; Figure 3G; F_(2,9)_ = 11.73, *P* = .003; Figure 3H), which signified that the synthesis of GABA was reduced. At the same time, the levels of surface α5GABA_A_Rs were up‐regulated in the laparotomy mice (F_(2,12)_ = 6.56, *P* = .01; Figure 3I). These results demonstrate that anesthesia and surgery could disrupt the GABAergic system in the hippocampus and selectively increase expressions of surface α5GABA_A_Rs.

### 3.4 Pharmacological blockade of α5GABA_A_Rs with L655,708 could reverse anesthesia and surgery induced hippocampus‐dependent memory deficits in aged mice

**Figure 4:**
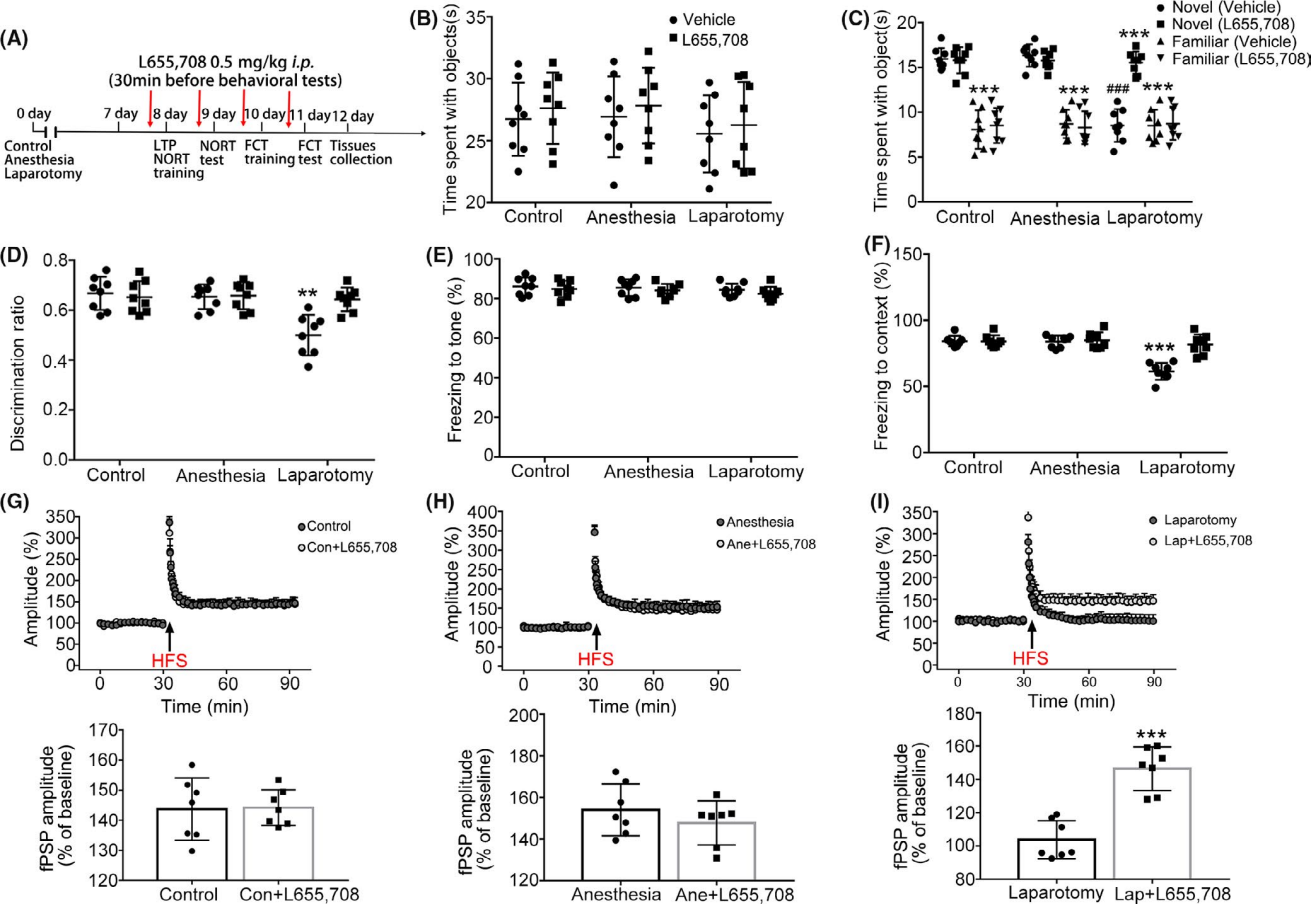
L655,708 could reverse anesthesia and surgery induced learning and memory deficits in aged mice. A, The diagram shows the process of the experiment. The time points of L655,708 (0.5 mg/kg, *i.p*.) or vehicle administered are marked by the red arrow. Samples were taken at the end of the experiment. B‐D, In the NORT, the time spent with objects was similar among the three groups, while the time spent with a novel object and the discrimination ratio were increased in the laparotomy mice after using L655,708 (n = 8). E‐F, In the FCT, there was no difference in the tone freezing time after using L655,708. However, the freezing scores for memory of context were increased in the laparotomy mice after using L655,708 (n = 8). G‐I, The amplitude of fPSPs in the laparotomy group was increased after using L655,708, while there was no difference in the control and anesthesia mice (n = 7). Data are presented as mean ± SEM. **P < .01, ***P < .001, ^###^ *P* < .001

To further investigate the role of α5GABA_A_Rs after anesthesia and surgery in inducing learning and memory deficits, the specific inverse agonist L655,708 was used to reduce the affinity for GABA by acting upon the α5GABA_A_Rs. In the NORT, no significant difference was found in the total time spent on identical sample objects during the training stage after using L655,708 (F_(2,14)_ = 0.003, *P* = .99; Figure 4B). However, the time spent exploring the novel object and the discrimination ratio were prominently increased in the laparotomy group after administering L655,708 (F_(6,42)_ = 14.34, *P* < .001; Figure 4C; F_(2,14)_ = 8.06, *P* = .005; Figure 4D). In the FCT, no difference was found in the freezing time to the tone (F_(2,14)_ = 0.03, *P* = .97; Figure 4E). The percentage of context freezing time was increased in the laparotomy mice after administering L655,708 (F_(2,14)_ = 29.82, *P* < .001; Figure 4F). In addition, the amplitude of fPSPs in the laparotomy mice was increased from 103.8% ± 4.3% to 146.4% ± 4.9% after the application of L655,708 (t = 6.47, *P* < .001; Figure 4I), and there was no difference between the control and anesthesia groups (t = 0.11, *P* = .92; Figure 4G; t = 1.02, *P* = .33; Figure 4H). These results indicate that blocking α5GABA_A_Rs with L655,708 could reverse anesthesia and surgery induced hippocampus‐dependent memory deficits.

### 3.5 P38 MAPK signaling pathway was specifically activated after anesthesia and surgery in aged mice

**Figure 5:**
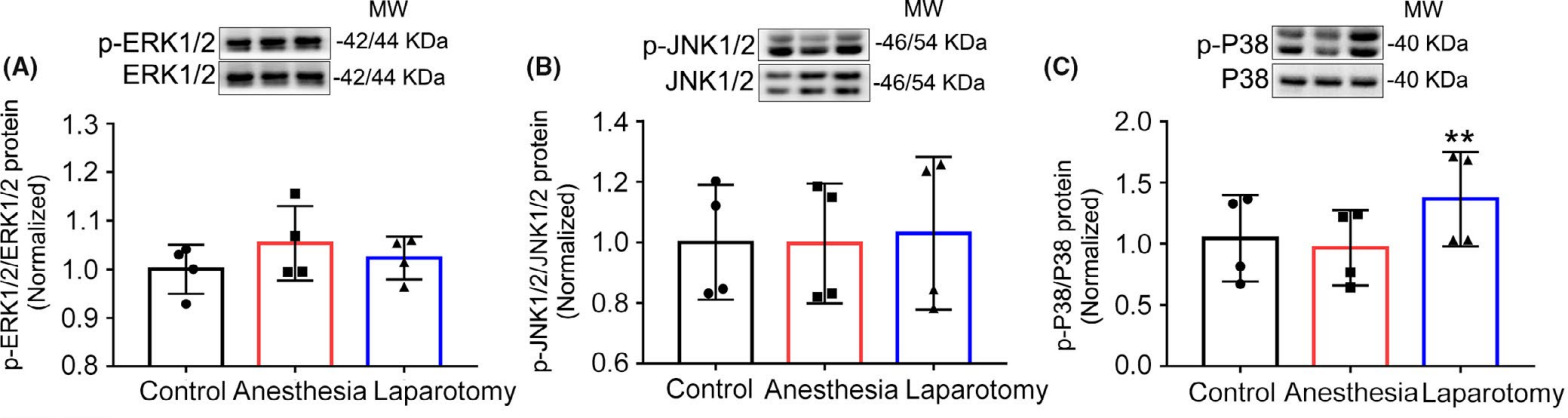
The protein levels of MAPK signaling pathway in the hippocampus. A‐C, The protein level of p‐P38 was increased after laparotomy compared to the control and anesthesia groups, and there was no difference in the expressions of P38, JNK1/2, p‐JNK1/2, ERK1/2, and p‐ERK1/2 (n = 4). Data are presented as mean ± SEM. **P < .01

To explore the potential signaling pathway of the cellular response to inflammatory stimuli, the expressions of MAPK signaling pathways including P38, p‐P38, JNK1/2, p‐JNK1/2, ERK1/2, and p‐ERK1/2 proteins were evaluated using Western blot. The expression of p‐P38 was obviously up‐regulated in the laparotomy group (F_(2,9)_ = 1.45, *P* = .28; Figure 5C). No statistical difference was observed in the expression of P38, ERK1/2, p‐ERK1/2, JNK1/2, and p‐JNK1/2 (F_(2,9)_ = 2.83, *P* = .12; Figure 5A; F_(2,9)_ = 0.03, *P* = .97; Figure 5B). These results indicate that the P38 MAPK signaling pathway was specially activated in the hippocampus after anesthesia and surgery in aged mice.

### 3.6 Pharmacological blockade of the P38 MAPK signaling pathway with SB203,580 could reverse anesthesia and surgery induced hippocampus‐dependent memory deficits in aged mice

**Figure 6:**
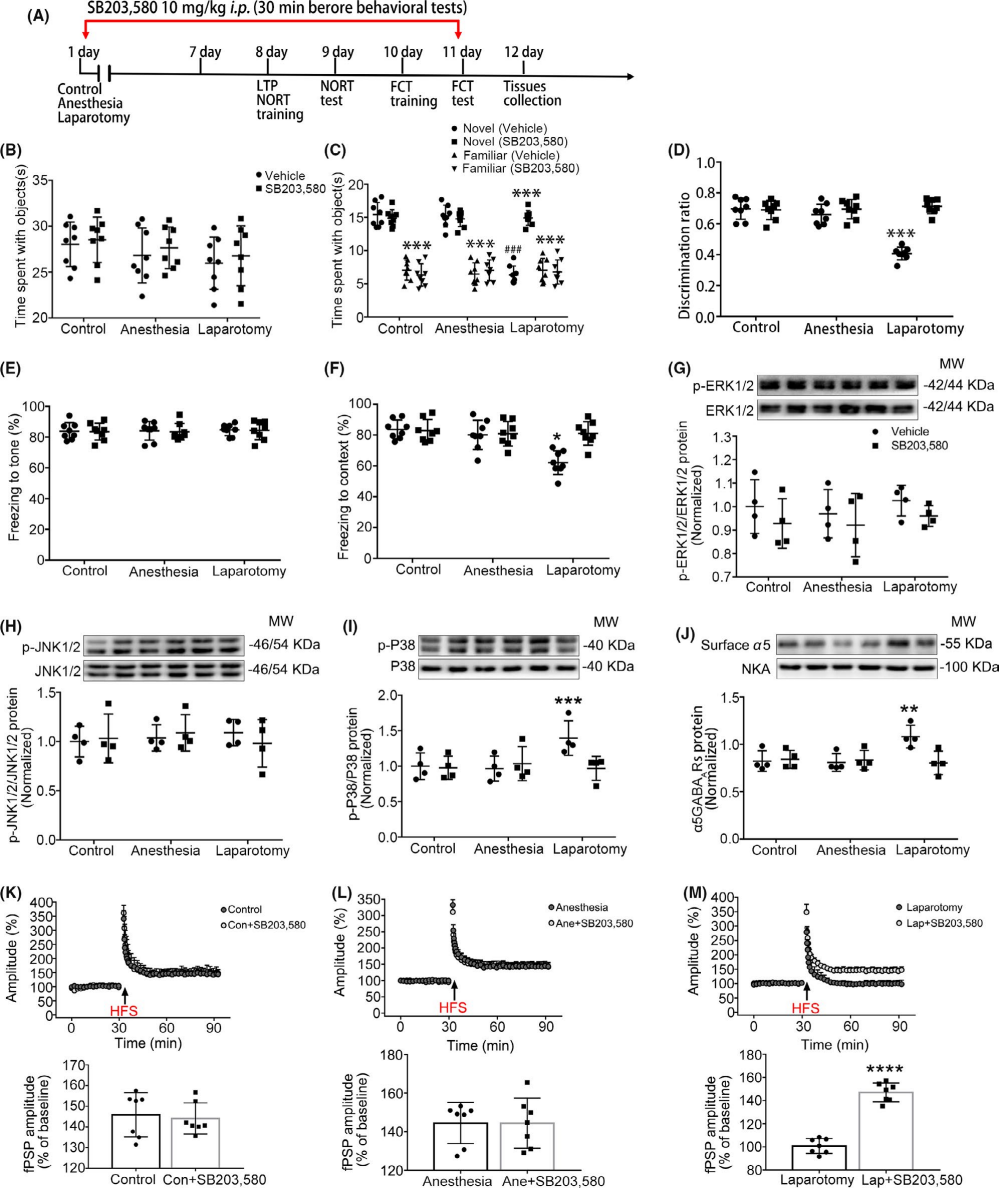
SB203,580 could reverse anesthesia and surgery induced learning and memory deficits in aged mice. A, The diagram shows the process of the experiment. The time points of SB203,580 (10 mg/kg *i.p*.) or vehicle administered are marked by the red arrow. Samples were taken at the end of the experiment. B‐D, In the NORT, the time spent with objects was similar among the three groups, while the time spent with the novel object and the discrimination ratio were increased in the laparotomy mice after using SB203,580 (n = 8). E‐F, In the FCT, the context freezing time was increased in the laparotomy mice after using SB203,580, and there was no difference in the tone freezing time (n = 8). G‐J, The protein levels of p‐P38 and surface α5GABA_A_Rs were decreased in the laparotomy mice after using SB203,580, and no difference was found in the expressions of p‐JNK1/2 and p‐ERK1/2 (n = 4). K‐M, The amplitude of fPSPs in the laparotomy mice was increased after using SB203,580, and there was no difference in the control and anesthesia mice (n = 7). Data are presented as mean ± SEM. *P < .05, **P < .01, ***P < .001, ^###^ *P* < .001, ****P < .0001

SB203,580 is the selective inhibitor of the P38 MAPK signaling pathway. Therefore, we used SB203,580 to further investigate the role of the P38 MAPK signaling pathway in inducing learning and memory deficits after anesthesia and surgery. In the NORT, no difference was found in the total time spent exploring identical sample objects among the three groups after using SB203,580 (F_(2,14)_ = 0.01, *P* = 0.99; Figure 6C). However, the time spent at the novel object and the discrimination ratio were prominently increased in the laparotomy group after administering SB203,580 (F_(6,42)_ = 28.08, *P* < .001; Figure 6C; F_(2,14)_ = 166, *P* < .001; Figure 6D). In the FCT, no statistical difference was found in the freezing time to the tone (F_(2,14)_ = 0.09, *P* = .91; Figure 6E), while the percentage of context freezing time was increased in the laparotomy group after administering SB203,580 (F_(2,14)_ = 6.03, *P* = .01; Figure 6F). At the same time, a qualitative decrease in p‐P38 and surface α5GABA_A_R expressions was observed in the laparotomy mice after using SB203,580 (F_(2,6)_ = 10.38, *P* = .01; Figure 6I; F_(2,6)_ = 35.4, *P* = .005; Figure 6J), but there was no difference shown in the expressions of p‐ERK1/2 and p‐JNK1/2 (F_(2,6)_ = 1.11, *P* = .39; Figure 6G; F_(2,6)_ = 3.87, *P* = .08 Figure 6H). In hippocampal slices, the amplitude of fPSPs in the laparotomy mice was increased from 100.7% ± 2.4% to 147.1% ± 3.1% after the application of SB203,580 (t = 11.79, *P* < .0001; Figure 6M), yet there was no difference between the control and anesthesia groups (t = 0.32, *P* = .75; Figure 6K; t = 0.01, *P* = .99; Figure 6L). These results illustrate that blocking the P38 MAPK signaling pathway could reverse anesthesia and surgery induced hippocampus‐dependent memory deficits possibly by preventing the trafficking of α5GABA_A_Rs.

## 4 DISCUSSION

PND are mainly experienced as memory deficits by elderly people which seriously affects their quality of life, but the specific pathophysiology mechanisms remain unclear. In the current study, we found that anesthesia and surgery caused robust neuroinflammation in the hippocampus, which in turn disrupted the GABAergic system, especially by targeting surface α5GABA_A_Rs traffic through activating the P38 MAPK signaling pathway which eventually led to hippocampus‐dependent memory deficits.

Numerous studies have shown that neuroinflammation is the main reason for PND.^9^, ^25^ Systemic inflammation caused by surgery could induce neuroinflammation, mainly through destroying the permeability of the blood‐brain barrier,^26^, ^27^, ^28^ hence, promoting the activation of local microglia. Activated microglia subsequently release more inflammatory cytokines.^9^, ^25^, ^29^, ^30^, ^31^ In our research, the levels of IL‐1β, IL‐6, and TNF‐α in the hippocampus were up‐regulated and microglia clearly activated after anesthesia and surgery. The results indicate that the hippocampus suffered significant inflammation after laparotomy under isoflurane anesthesia. However, TNF‐α was also increased after anesthesia without surgery, but no activation of microglia was found in the hippocampus. It suggests that isoflurane anesthesia alone could not induce harmful inflammation in the hippocampus, which is in line with Wang et al and Kawano et al’s findings.^32^, ^33^ Callaway et al^34^ and Crosby et al^35^ demonstrated that exposure to sevoflurane or isoflurane alone had no impact on learning and memory in the rodent. Walters et al^36^ also reported that learning task performance showed no significant changes after exposure to anesthesia alone in adult populations. In brief, hippocampal neuroinflammation caused by anesthesia and surgery was much more serious in aged mice than that caused by anesthesia alone. The degree of severity of hippocampal neuroinflammation could be closely related to the memory loss after anesthesia and surgery.

In the central nervous system, the GABAergic system contributes to controlling the excitability of neuronal networks. However, the functions of the GABAergic system are easily affected by inflammation, including GABAergic neuronal density, GABA, and its synthetic machinery and GABA receptors. Qiu et al^37^ reported that hippocampal parvalbumin interneurons contributed to cognitive dysfunction in aged mice. Here, we found that the concentration of GABA in the hippocampus was decreased after anesthesia and surgery. At the same time, the protein expressions of GAT‐3 and GAD65^38^ were decreased after anesthesia and surgery. Dysfunction of GAT‐3 is related to several neurological diseases, such as Alzheimer's disease.^39^ Other studies showed that GAD65 is associated with GABAergic synaptic transmission and plasticity, and that the reduction in GAD65 contributed to neuropsychiatric disorders in mice.^40^ Here, we found that transcription of the α5 subunit and the levels of surface α5GABA_A_Rs were increased after anesthesia and surgery. Sustained increase in α5GABA_A_Rs activity disrupted memory and synaptic plasticity.^41^ Pharmacologically blocking of α5GABA_A_Rs with L655,708 could reverse hippocampus‐dependent memory deficits and LTP. Inhibition or elimination of α5GABA_A_Rs improved the Morris water maze performance and fear conditioning in mice.^42^ However, Gao et al^43^ suggested that prophylactic use of L655,708 does not prevent isoflurane induced memory deficits in aged mice. One reason could be that they used a different animal model. They took an animal model which only received inhalation anesthesia, without surgery, whereas in our study, the animal received both inhalation anesthesia and surgery. The pathophysiology process could therefore be different between these two animal models. The other reason could be that L655,708 was administrated prophylactically in their study, but postanesthesia and postsurgery in ours.

Up‐regulation of surface α5GABA_A_Rs is primarily associated with activation of the P38 MAPK signaling pathway, and the signaling pathway is known to be an important regulator of GABA_A_Rs trafficking.^44^ Cytokines, that induce activation of the P38 MAPK signaling pathway, are widely reported in some other inflammation models.^45^ In our study, we tested three typical pathways of MAPK and found that the protein level of p‐P38 selectively increased. Pharmacological blocking of the P38 MAPK signaling pathway with SB203,580 reversed anesthesia and surgery induced hippocampus‐dependent memory deficits, and reduced the levels of p‐P38 and surface α5GABA_A_Rs.

There are several limitations in our study. Firstly, we did not explore the changes in tonic inhibitory currents regulated by α5GABA_A_Rs to investigate the effect of α5GABA_A_Rs on postsynaptic functions. Secondly, since the gene knockout technology can effectively distinguish the functions of different subunits, we could have used knockout mice to further verify the functions of α5GABA_A_Rs. Lastly, some studies have demonstrated that postoperative pain is also a factor influencing the cognitive behavior. Postoperative pain could not be totally avoided in this study and deserves further investigation.

In summary, our study revealed that hippocampus‐dependent memory was disrupted by anesthesia and surgery rather than by anesthesia alone. Anesthesia and surgery caused neuroinflammation in the hippocampus, which consequently disrupted the GABAergic system, increased the expressions of surface α5GABA_A_Rs especially through activating the P38 MAPK signaling pathway, which eventually led to dysfunctions of hippocampus‐dependent memory. Therefore, our research may provide a new viewpoint for exploring the mechanisms of PND, while α5GABA_A_Rs may serve as a potential target for preventing or treating PND.

## CONFLICTS OF INTEREST

The authors declare no competing interests.

## Supporting information

Table S1

Data S1
