# Supplementary figures and images for: Disruption of the GABAergic system contributes to the development of perioperative neurocognitive disorders after anesthesia and surgery in aged mice
Source: CNS Neurosci Ther. 2020 Jun 2;26(9):913–24. doi: 10.1111/cns.13388 (PMC7415208; doi:10.1111/cns.13388)

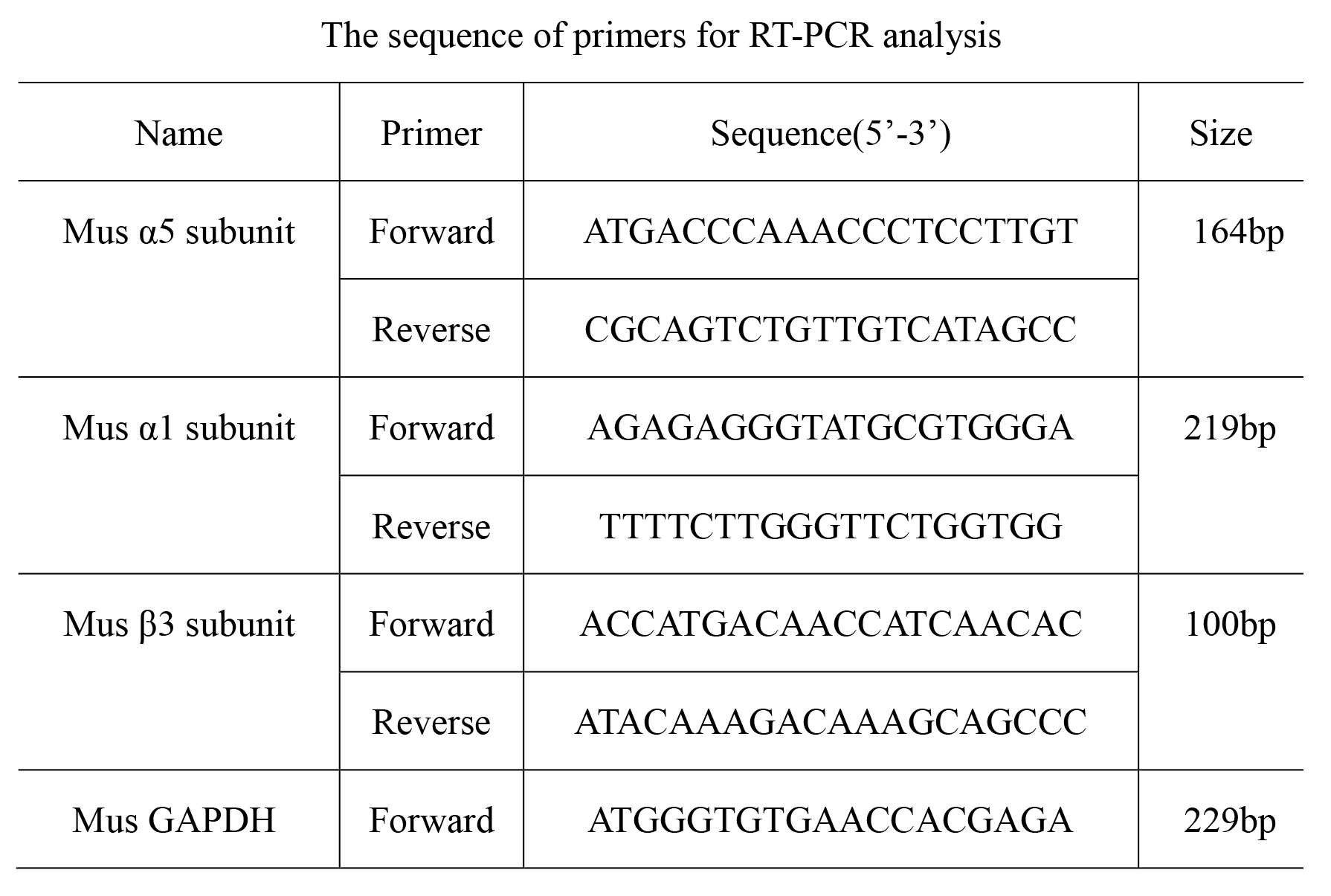

Supplement: Supplementary file 1 — Table S1 [file CNS-26-913-s001.tif]
