# Supplementary material for: Disruption of the GABAergic system contributes to the development of perioperative neurocognitive disorders after anesthesia and surgery in aged mice
Source: CNS Neurosci Ther. 2020 Jun 2;26(9):913–24. doi: 10.1111/cns.13388 (PMC7415208; doi:10.1111/cns.13388)
Supplement: Supplementary file 2 — Data S1 [file CNS-26-913-s002.pdf]

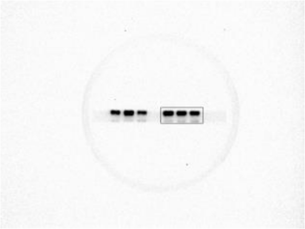

GAT-3 Full unedited blot for Figure3G

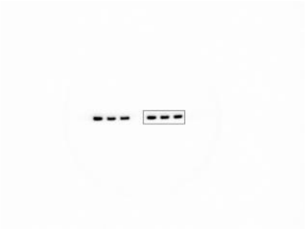

GAPDH Full unedited blot for Figure3G

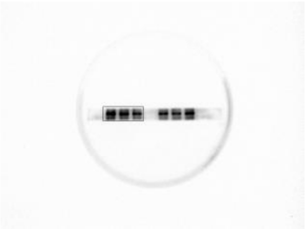

GAD65 Full unedited blot for Figure3H

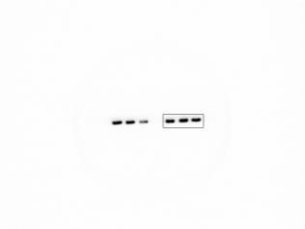

GAPDH Full unedited blot for Figure3H

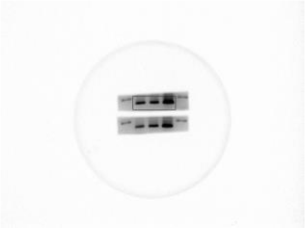

Surface α5GABA$_A$Rs Full unedited blot for Figure3I

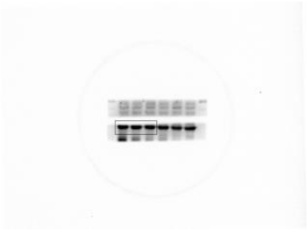

NKA Full unedited blot for Figure3I

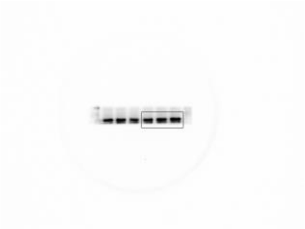

p-ERK1/2 Full unedited blot for Figure5A

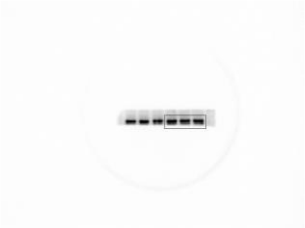

ERK1/2 Full unedited blot for Figure5A

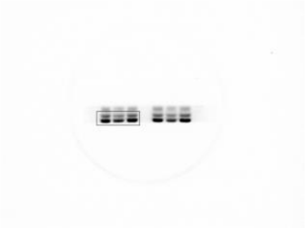

p-JNK1/2 Full unedited blot for Figure5B

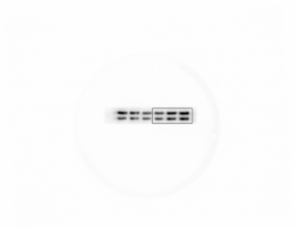

JNK1/2 Full unedited blot for Figure5B

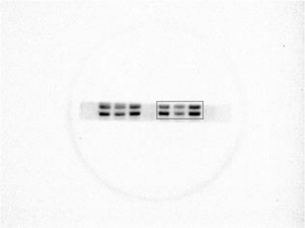

p-P38 Full unedited blot for Figure5C

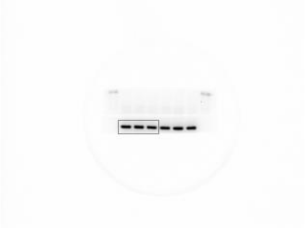

P38 Full unedited blot for Figure5C

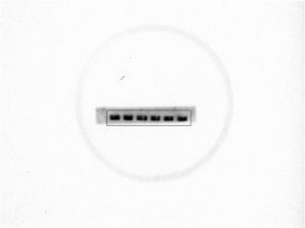

p-ERK1/2 Full unedited blot for Figure6G

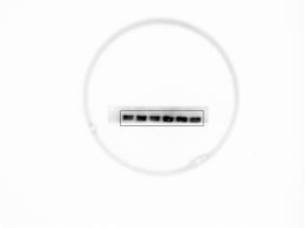

ERK1/2 Full unedited blot for Figure6G

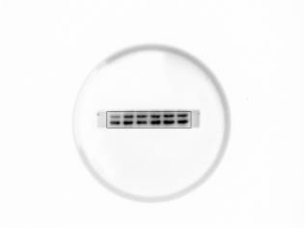

p-JNK1/2 Full unedited blot for Figure6H

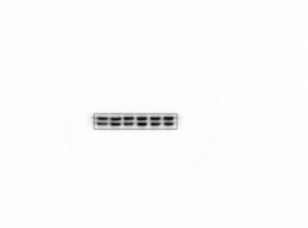

JNK1/2 Full unedited blot for Figure6H

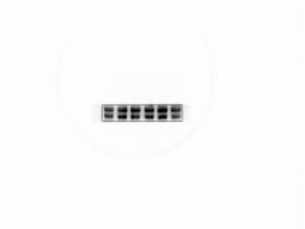

p-P38 Full unedited blot for Figure6I

P38 Full unedited blot for Figure6I

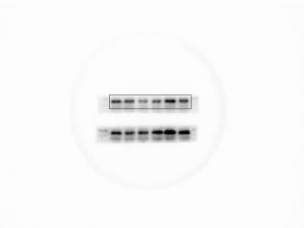

Surface α5$GABA_A$Rs Full unedited blot for Figure6J

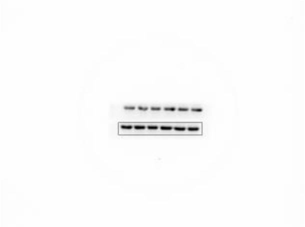

NKA Full unedited blot for Figure6J
